# Factors affecting patient safety culture in terms of compliance with preventing bloodborne pathogens among general hospital nurses

**DOI:** 10.1186/s12912-020-00529-4

**Published:** 2021-01-04

**Authors:** Na Young Kim, Kyoung Ja Moon

**Affiliations:** College of Nursing, Keimyung University, Daegu, 42601 South Korea

**Keywords:** Patient safety, Patient safety culture, Bloodborne pathogen, Nurses

## Abstract

**Background:**

The present study aims to investigate the relationship between patient safety culture and the prevention of transmitting bloodborne pathogens among nurses at a general hospital.

**Methods:**

The participants were 284 nurses working at a general hospital located in a city, and the data were collected between April 26 and May 15, 2019. Questionnaires on patient safety culture and the prevention of bloodborne pathogens were used, and SPSS version 22.0 was used for descriptive and hierarchical regression analysis.

**Results:**

The results showed that the following factors affected the prevention of bloodborne pathogens: experience with needle stick and sharps injuries (β = − 0.94), teamwork (β = 0.41), knowledge and attitude toward patient safety (β = 0.34), leadership (β = 0.15), and priority of patient safety (β = 0.14). The model’s explanatory power was 53% (*F* = 32.26, *p* =< 0.001).

**Conclusions:**

To increase the compliance of general hospital nurses with practices that promote the prevention of bloodborne pathogens, it is necessary to actively prevent needle sticks and sharps injuries. It is also necessary to prioritize patient safety and to develop and verify the effects of various programs that emphasize factors of patient safety culture, such as leadership, teamwork, knowledge, and attitude.

## Background

As the standard of living has improved and the provision of medical services and public interest in health have increased, patients and caregivers who use hospitals have increasingly demanded high-quality medical services in safe environments [1]. Access to appropriate medical services is regarded as a basic patient right [2]; however, the increasing severity of patient conditions and complexity of medical services still threaten patient safety. Improving patient safety and the quality of care has become a critical public health issue [3, 4].

Patient safety policies aim to minimize potential risks in medical services to prevent errors and to minimize risk factors, with the ultimate goal of decreasing the incidence of medical malpractice [5, 6]. Incidents of medical malpractice cause financial loss, distrust in healthcare professionals and hospitals, and longer hospital stays, directly influencing patient health and lives; therefore, medical malpractice is an important factor that decreases the quality of care [7, 8]. The American Institute of Medicine [9] emphasized the need to mitigate medical malpractice and suggested fostering a culture that prioritizes patient safety.

Patient safety culture is an organizational culture in which hospital staff recognize patient safety as a priority. This is guided not only by patient safety policies and improved organizational systems but also by the management of patient safety through active communication and teamwork between the members [10]. Nurses’ perceptions of patient safety culture have been seen to increase their compliance with safe nursing practices, thus creating a safe medical environment [11]. Nurses often encounter situations requiring them to make critical judgments and independent decisions and where factors such as teamwork, knowledge, techniques, attitude, leadership, and communication are necessary for efficient workflow and problem solving [12].

Among the various healthcare-associated infections (HAIs), bloodborne infections are caused by exposure to blood or bodily fluids of a patient. Although prevention is of the utmost importance, when such exposure occurs, team members often criticize one another instead of communicating effectively, which creates obstacles to patient safety [11, 13]. Nurses who provide direct care to patients are at high risk of exposure to bloodborne infections such as hepatitis B, hepatitis C, and HIV [14–16].

According to previous studies, patient safety and patient safety culture have a positive influence on outcomes [17], and the application of various interventions could change the organizational safety culture and improve infection control [18]. Therefore, it is necessary to develop interventions for infection control by investigating the factors affecting patient safety culture for bloodborne pathogen prevention.

## Methods

### Study design

This cross-sectional study aims to investigate the relationship between patient safety culture and compliance with bloodborne pathogen prevention in nurses of a general hospital.

### Setting & participants

Participants were selected through convenience sampling and included nurses working at Pohang General Hospital (which is located in Pohang city and has more than 500 beds) who could understand and answer the survey questions voluntarily.

The sample size was calculated on G*Power 3.1.9.2, which revealed that a minimum of 256 participants was needed to perform a regression analysis with a median effect size of 0.15, significance level of 0.05, statistical power of 0.95, and 29 predictors. To account for a 15% drop-out rate, the questionnaires were distributed to 294 participants. A total of 284 questionnaires were collected, and the response rate was 97%.

### Measurement tools

#### Patient safety culture

The Korean Patient Safety Culture Survey Instrument for Hospitals developed by Lee (2015) [19] is used to measure patient safety culture. The tool consists of 35 questions in seven domains. The questions are divided into three dimensions: organization, department, and individual. The subdomains of the organization dimension include questions on leadership (9), patient safety policy and procedures (4), and patient safety improvement systems (4). The subdomains of the department dimension include questions on teamwork (6) and nonpunitive environments (4). The subdomains of the individual domain comprise questions on knowledge and attitude toward patient safety (5) and the priority of patient safety (3). Each question is rated on a 5-point Likert scale ranging from 1 (“strongly disagree”) to 5 (“strongly agree”). Higher scores indicate higher awareness of patient safety culture. In terms of reliability, Cronbach’s α was 0.93 in Lee (2015) [19], 0.93 in Sung (2018) [20], and 0.87 in the present study.

#### Compliance with bloodborne pathogen prevention

To measure compliance with practices promoting the prevention of bloodborne pathogens, the Compliance with Bloodborne Pathogens Prevention Scale developed by Lee (2017) [15] was used. The tool consists of 18 questions in four domains: compliance with standard precautions (8), compliance with infection exposure prevention when using instruments (3), emergency treatment after exposure to infections (3), and postexposure reporting and follow-up management (4). The questions are rated on a 4-point Likert scale: “never” = 1, “sometimes” = 2, “often” = 3, and “always” = 4. The total score ranges between 7 and 18; a higher total score indicates higher compliance with practices promoting the prevention of bloodborne pathogens. In terms of the reliability of the tool, Cronbach’s α was 0.88 in Lee (2017) [15] and 0.93 in the present study.

### Data collection

The data for this study were collected between April 26 and May 15, 2019, using structured self-reported questionnaires distributed to nurses working at Pohang General Hospital located in Pohang City. Prior to data collection, the director of nursing services granted approval by telephone. Subsequently, the researcher visited the nursing department to explain the study purpose and obtain approval for data collection. The purpose and methods of the study were explained in detail to the head nurses of the respective departments, and the questionnaires were explained and distributed in the nurses’ lounge of each department. Nurses who wished to participate were then asked to complete the questionnaires. The researcher visited the participants in their respective departments during each shift to explain the purpose of the study, the methods involved, and how the questionnaire should be completed. Participants were assured that the collected data would be used solely for research purposes, their identities would remain anonymous, and they could withdraw their participation at any point. The nurses who provided voluntary consent were asked to complete the questionnaires, and the completed questionnaires were collected by the researcher.

### Statistical methods

The collected data were analyzed using SPSS/WIN 22.0 software. The demographic characteristics, patient safety culture, and compliance with bloodborne pathogen prevention were analyzed using frequency, percentage, mean, and standard deviation. The difference in bloodborne pathogen prevention according to the general characteristics of the subjects was analyzed by t-test and one-way ANOVA, and a post hoc test was performed by the Scheffé test. The relationship between patient safety culture and compliance with practices promoting the prevention of bloodborne pathogens was assessed using the Pearson correlation coefficient. A hierarchical multiple regression analysis was conducted to investigate the factors that influence the participants’ compliance with practices promoting the prevention of bloodborne pathogens.

### Ethical considerations

The present study was approved by the Institutional Review Board (IRB, 40525–201901-HR-126-02) at Keimyung University in Deagu City. The participants provided written informed consent after the study purpose was explained to them. The consent form outlined that participation is voluntary, participant anonymity will be protected, the participants may withdraw their participation whenever they desire with no repercussions, and the collected data will be used solely for research purposes.

## Results

Table 1 shows the demographic and clinical characteristics of the participants. It was noted that 275 (96.8%) participants were female, and 202 (71.1%) were below 30. Of the total participants, 205 (72.1%) had a bachelor’s degree and 234 (82.4%) were staff nurses. In terms of total work experience, 199 (70.1%) had less than 5 years of experience, and 85 (29.9%) had more than 5 years. Further, 234 (82.4%) were noted to have been working in their current unit for less than 5 years, and 50 (17.8%) had been working in their current unit for more than 5 years. Of the total, 55 (19.4%) worked in non-surgical internal medicine units. In terms of experiencing needle stick and sharp injury (NSSI), 221 (77.8%) had experienced NSSI, in contrast with 63 (22.2%) who had not. Further, 37 (58.7%) experienced NSSI from disposable syringes, and 21 (33.3%) experienced NSSI while disassembling needles or sharp instruments. It was noted that 253 (89.1%) never experienced mucous membrane exposure to blood and bodily fluids, and 249 (87.7%) never experienced skin exposure to blood and bodily fluids. Of the total, 257 (90.5%) never completed a report on infection exposure, and 231 had received the hepatitis B vaccine (81.3%). The results showed that 142 completed 0 patient safety case reports (50%), and 135 (47.5%) completed 1–5 patient safety case reports. Further, 259 (91.2%) participants received education on prevention of bloodborne pathogens, and 119 (41.9%) and 123 (43.3%) received education at hospital and at both school and hospital, respectively.

**Table 1:** Demographic and clinical characteristics of the subjects (*N* = 284)

Characteristics	Categories	*n* (%)	
Sex	Female	275	(96.8)
	Male	9	(3.2)
Age group	< 30	202	(71.1)
	≥30	82	(28.9)
Education level	Diploma	72	(25.4)
	Bachelor’s	205	(72.1)
	>Master’s	7	(2.5)
Position	Staff nurse	234	(82.4)
	Charge nurse	31	(10.9)
	Head nurse	19	(6.7)
Work experience	≤5	199	(70.1)
	> 5	85	(29.9)
Current unit employed	≤5	234	(82.4)
	> 5	50	(17.6)
Work unit	Medicine (nonsurgical)	55	(19.4)
	Surgery	52	(18.3)
	Intensive care unit	46	(16.2)
	Emergency	30	(10.6)
	Operating room	27	(9.5)
	Women	7	(2.5)
	Others	67	(23.5)
Experienced NSSI for last 1 year	Yes	63	(22.2)
	No	221	(77.8)
Type of devices causing NSSI	Disposable syringe	37	(58.7)
	Blood glucose lancet	14	(22.2)
	Other sharp device	12	(19.1)
Procedures causing NSSI	Inserting a needle	5	(7.9)
	Disposing of used items	10	(15.9)
	Recapping a needle	8	(12.7)
	Disassembling needle or sharp instrument	21	(33.3)
	Others	19	(30.2)
Blood and body fluid exposure experience of mucous membrane	Yes	31	(10.9)
	No	253	(89.1)
Blood and body fluid exposure experience of skin	Yes	35	(12.3)
	No	249	(87.7)
Report infection exposure	Yes	27	(9.5)
	No	257	(90.5)
Vaccination for hepatitis B	Yes	231	(81.3)
	No	30	(10.6)
	Do not know	23	(8.1)
Number of patient safety case reports	0	142	(50)
	1∼5	135	(47.5)
	6≤	7	(2.5)
Education experience with bloodborne pathogen prevention	Yes	259	(91.2)
	No	25	(8.8)
Educational institution for Bloodborne pathogen prevention	College	17	(6.0)
	Hospital	119	(41.9)
	Both	123	(43.3)
	Others	25	(8.8)

*NSSI* Needlestick and sharps injuries

According to the participants’ demographic characteristics, compliance with practices promoting prevention of bloodborne pathogens was higher in participants older than 30 (*F* = − 2.04, *p* = 0.042), head nurses and those in higher positions (*F* = 3.89, *p* = 0.021), and participants without NSSI experience (*t* = − 2.29, *p* = 0.024) (Table 2).

**Table 2:** Compliance with bloodborne pathogen prevention according to subject characteristics (*N* = 284)

Variables	Categories	Compliance of bloodborne pathogen prevention		
		M ± SD	t/F	*p*
Sex	Female	3.40 ± 0.40	0.49	0.631
	Male	3.27 ± 0.75		
Age group	< 30	3.41 ± 0.42	−2.04	0.042
	≥30	3.53 ± 0.42		
Education level	Diploma	3.44 ± 0.45	0.75	0.474
	Bachelor’s	3.38 ± 0.39		
	>Master’s	3.34 ± 0.52		
Position	Staff nurse	3.43 ± 0.43	3.89	0.021 a<c^*^
	Charge nurse	3.47 ± 0.42		
	<Head nurse	3.70 ± 0.34		
Work experience	≤5	3.42 ± 0.42	−1.21	0.227
	> 5	3.49 ± 0.42		
Current unit employed	≤5	3.44 ± 0.42	−0.48	0.627
	> 5	3.47 ± 0.44		
Work unit	Medicine	3.41 ± 0.37	0.60	0.729
	Surgery	3.34 ± 0.44		
	Intensive care unit	3.46 ± 0.40		
	Emergency	3.33 ± 0.40		
	Operating room	3.42 ± 0.50		
	Women	3.52 ± 0.29		
	Others	3.39 ± 0.41		
Experienced NSSI for last 1 year	Yes	3.33 ± 0.49	−2.29	0.024
	No	3.48 ± 0.39		
Type of devices causing NSSI	Disposable syringe	3.33 ± 0.50	0.14	0.868
	Blood glucose lancet	3.31 ± 0.38		
	Other sharp device	3.25 ± 0.61		
Procedures causing NSSI	Inserting a needle	3.43 ± 0.44	1.14	0.347
	Disposing of a needle	3.57 ± 0.66		
	Recapping a needle	3.19 ± 0.67		
	Disassembling needle or sharp instrument	3.20 ± 0.38		
	Others	3.31 ± 0.41		
Blood and body fluid exposure experience of mucous membrane	Yes	3.47 ± 0.51	1.05	0.293
	No	3.38 ± 0.40		
Blood and body fluid exposure experience of skin (1 year)	Yes	3.34 ± 0.40	−0.77	0.445
	No	3.40 ± 0.41		
Report infection exposure	Yes	3.29 ± 0.35	−1.42	0.157
	No	3.40 ± 0.42		
Vaccination of hepatitis B	Yes	3.40 ± 0.40	0.17	0.848
	No	3.37 ± 0.42		
	Do not know	3.36 ± 0.53		
Number of patient safety case reports	0	3.38 ± 0.42	1.16	0.314
	1∼5	3.41 ± 0.40		
	6≤	3.18 ± 0.43		
Education experience for bloodborne pathogen prevention	Yes	3.40 ± 0.41	0.98	0.327
	No	3.32 ± 0.43		
Educational institution for bloodborne pathogen prevention	College	3.20 ± 0.44	1.74	0.160
	Hospital	3.41 ± 0.42		
	Both	3.41 ± 0.39		
	Others	3.37 ± 0.25		

Sheffe test

A significant correlation was found between awareness of patient safety culture and compliance with bloodborne pathogen prevention (*r* = 0.36, *p* < 0.010). In terms of sub-categories, leadership had the highest correlation (*r* = 0.85, *p* < 0.010), followed by teamwork (*r* = 0.82, *p* < 0.010), patient safety policy/procedures (*r* = 0.77, *p* < 0.010), knowledge and attitude toward patient safety (*r* = 0.76, *p* < 0.010), system to improve patient safety (*r* = 0.61, *p* < 0.010), and non-punitive environment (*r* = 0.17, *p* < 0.050). Priority of patient safety, which was also a sub-category of patient safety culture awareness did not show a significant correlation (*r* = 0.07, *p* = 0.223) (Table 3).

**Table 3:** Correlations of patient safety culture and bloodborne pathogen prevention (*N* = 284)

Variables	Patient safety culture	Subcategory							Compliance with bloodborne pathogen prevention
		Leadership	Teamwork	Knowledge /attitude	Policy /procedures	Nonpunitive responses	Improvement system	Prioritized	
	r(*p*)	r(*p*)	r(*p*)	r(*p*)	r(*p*)	r(*p*)	r(*p*)	r(*p*)	r(*p*)
Patient safety culture	1								
Leadership	.85(<.010)	1							
Teamwork	.82(<.010)	.52(<.010)	1						
Knowledge/attitude	.76(<.010)	.37(<.010)	.30(<.010)	1					
Policy/procedures	.77(<.010)	.58(<.010)	.39(<.010)	.36(<.010)	1				
Nonpunitive responses	.17(<.010)	.07(.192)	.10(.066)	−.01(.812)	.13(<.050)	1			
Improvement Safety system	.61(<.010)	.41(<.010)	.29(<.010)	.26(<.010)	.49(<.001)	.11(.062)	1		
Prioritized	.07(.223)	.14(<.050)	.07(.188)	.13(<.050)	.14(<.050)	.43(<.010)	.11(.053)	1	
Compliance with bloodborne pathogen prevention	.36(<.010)	.48(<.010)	.59(<.010)	.53(<.010)	.38(<.001)	.13(<.050)	.26(<.010)	.26(<.010)	1

A hierarchical multiple regression analysis was conducted to identify factors that influence compliance with bloodborne pathogen prevention. In the analysis, job position (demographic characteristic) and experience of NSSI (clinical characteristic) were used as the dummy variables, and patient safety culture and its sub-categories were used as independent variables. Model 1, which included job position, explained 1% (*F* = 4.26, *p* = 0.040) of compliance with practices promoting prevention of bloodborne pathogens, and the explanatory power increased by 2% in Model 2, which included NSSI experiences (*F* = 4.79, *p* = 0.009). In Model 3, the following sub-categories of patient safety culture awareness were found to have significant effects on compliance with bloodborne pathogen prevention: leadership (β = 0.15), teamwork (β = 0.41), knowledge and attitude toward patient safety (β = 0.34), and priority of patient safety (β = 0.14). The model had an explanatory power of 53% (*F* = 32.26, *p* =< 0.001) (Table 4).

**Table 4:** Factors influencing compliance with bloodborne pathogen prevention (*N* = 284)

Variables		Model 1		Model 2		Model 3	
		β(*p*)		β(*p*)		β(*p*)	
Demographic	Job position^a^	−.12	(.040)	−.10	(.092)	−.67	(.119)
Clinical-related	Sharp injured			−.14	(.023)	−.94	(.029)
Patient safety culture						−.85	(.466)
	Leadership					.15	(.031)
	Teamwork					.41	(<.001)
	Knowledge/attitude					.34	(<.001)
	Policy/procedures					.04	(.494)
	Nonpunitive approach					.57	(.311)
	Safety system					−.00	(.956)
	Prioritized					.14	(.005)
F(*p*)		4.26	(.040)	4.79	(.009)	32.26	(<.001)
R^2^		.02		.03		.54	
Adjusted R^2^		.01		.03		.53	

^a^Dummy variables: Job position (Staff nurse & Charge nurse 1, Nursing unit manager 0), Sharp injury (yes 1, no 0)

## Discussion

The present study analyzed the relationship between patient safety culture and nurses’ compliance with bloodborne pathogen prevention. When the participants’ compliance with the prevention of bloodborne pathogens was analyzed according to their demographic and clinical characteristics, compliance was found to be higher among head nurses, those in higher positions, and participants who had not experienced a needlestick and sharps injury (NSSI) in the preceding year. This finding was in contrast with those of previous studies that found no difference in compliance with prevention of bloodborne pathogens based on job position. However, the finding can be compared to those of previous research that reported that compliance was higher among those who had not experienced an NSSI than among those who had experienced an NSSI [16, 21, 22]. At the hospital where this study was conducted, head nurses and nurses in higher positions are responsible for the overall management of the unit, including monitoring of infection control, instead of providing direct care to patients; this might explain the results indicating higher awareness of infection prevention practices among higher-ranking nurses than among staff nurses. Thus, to improve compliance with the prevention of bloodborne pathogens, education on NSSIs should be improved, and continuous monitoring of prevention compliance should be implemented.

A significant correlation was seen between the participants’ patient safety culture and compliance with the prevention of bloodborne pathogens. In terms of patient safety culture, the subcategories of leadership, teamwork, patient safety policy, procedures, knowledge or attitude toward patient safety, patient safety improvement system, and priority of patient safety were significantly correlated, whereas nonpunitive responses did not correlate with compliance with prevention of bloodborne pathogens. Although direct comparisons are difficult to make due to the limited number of similar studies, one study reported that perception of patient safety culture in hospital nurses was significantly related to compliance with standard precautions [23]. Thus, as shown in the present study, since patient safety culture, including standard precautions, can influence the prevention of bloodborne pathogens, it is necessary to understand and pay attention to patient safety culture and relevant factors to prevent bloodborne infections [24].

According to a hierarchical regression analysis of factors that influence compliance with bloodborne pathogen prevention, experience with NSSIs and patient safety culture were found to be significant factors. Among the subcategories of patient safety culture, the significant factors were found to be teamwork, knowledge and attitude toward patient safety, leadership, and priority of patient safety.

First, participants who had never experienced an NSSI were found to be more compliant with bloodborne pathogen prevention than those who had experienced an NSSI. This finding is in line with that of a previous study that reported higher compliance with practices promoting bloodborne infection control among those who had never experienced blood exposure [16]. Moreover, our finding also agrees with that of another study reporting higher compliance with standard precautions among those who had never experienced needle stick injuries [25]. This seems to be the case because nurses’ compliance with practices that protect them from infection would have prevented NSSIs, and the finding further indicates the importance of preventing exposure to blood. Therefore, cases of bloodborne pathogen infections should be introduced in infection prevention training to emphasize the seriousness of the situation, and efforts should be made to help nurses habitually perform practices promoting the prevention of infections. Moreover, nurse awareness should be improved to encourage them to prioritize their own protection when providing care in situations where bloodborne infections are possible [21].

NSSI is a major cause of bloodborne infections, and considering that more than half of nurses who experience an NSSI encounter repeated injuries [26, 27], it is important to prevent NSSIs in the first place. Furthermore, to improve compliance with the prevention of bloodborne pathogens, continued safety training on the safe use of syringes and NSSIs is necessary. In particular, in the present study, NSSIs were seen to occur most often in the process of disassembling needles or sharp instruments, mostly while disposing and washing used instruments and while recapping used needles.

Therefore, these specific cases should be included in periodic infection education for nurses, and further training should be provided on precautions while using sharp instruments. In addition, appropriate administration or legislation should be implemented to prevent the recapping of used needles and to promote the wider use of safe injection devices [16, 28]. Furthermore, we found that nurses received more infection education at hospitals than during their regular nursing curriculum at school. Infection education programs should be a part of nursing curricula. It is also crucial to provide access to continuing education in clinical settings [29, 30].

Second, within patient safety culture, the subcategories of knowledge and attitude toward patient safety, leadership, and priority of patient safety were found to have positive effects on bloodborne pathogen prevention. This result is comparable to previous research findings that suggest that those with higher perceptions of patient safety culture have higher compliance with standard precautions [11] and compliance with practices promoting infection control [20]. These findings indicate that it is important to cultivate a positive patient safety culture through leadership and that patient safety culture is an important factor in the prevention of bloodborne pathogens. In terms of leadership in patient safety culture, a significant factor in the present study, it was found that leadership WalkRound programs aiming to change patient safety culture awareness inside and outside of South Korea have led to improvement and are a meaningful way to improve patient safety culture [31, 32].

The present study is significant because it confirms how patient safety culture influences compliance with practices promoting the prevention of bloodborne pathogens as a variable. In other words, the findings of the present study should be considered in developing programs for HAI prevention and training on infections. Moreover, this study used tools of Korean patient safety culture that were developed based on the consideration of Korean culture. A notable difference from the globally used Hospital Survey on Patient Safety Culture (HSOPSC) and Safety Attitudes Questionnaire (SAQ) [33–35] is the exclusion of questions from patient safety culture tools on the openness of communication, staff distribution, and handover. Instead, the Korean tools used in the present study added questions on policy or procedures, knowledge and attitude toward safety, and priority of patient safety [19]. The tools have different questions, probably because medical systems differ and the awareness of patient safety culture in Korea differs from that in other countries.

There are several limitations to this study: the data were collected through convenience sampling at a general hospital in one city, and it is difficult to generalize the findings due to regional bias. Moreover, the number of reports on patient safety incidents over the past year exceeded that of NSSI incidents because the hospital’s reporting system includes NSSIs as well as exposure to pressure ulcers, falls, and near misses.

## Conclusion

The present study analyzed the effects of hospital nurses’ patient safety culture on compliance with bloodborne pathogen prevention and found that an NSSI experience, leadership, and priority of patient safety were significantly related. Therefore, to increase the compliance of hospital nurses with practices promoting the prevention of bloodborne pathogens, it is necessary to carefully prevent NSSIs and to implement various programs that emphasize factors of patient safety culture, such as leadership, teamwork, and priority of patient safety.

## Data Availability

The datasets used and/or analysed during the current study available from the corresponding author on reasonable request.

## Abbreviations

HAI: Healthcare-associated infections
NSSI: Needlestick and sharps injury
HSOPSC: Hospital survey on patient safety culture
SAQ: Safety attitudes questionnaire
